# Factors Related to COVID-19 Preventive Behaviors: A Structural Equation Model

**DOI:** 10.3389/fpsyg.2021.676521

**Published:** 2021-07-05

**Authors:** Sanita Šuriņa, Kristine Martinsone, Viktorija Perepjolkina, Jelena Kolesnikova, Uku Vainik, Aleksejs Ruža, Jelena Vrublevska, Daria Smirnova, Konstantinos N. Fountoulakis, Elmars Rancans

**Affiliations:** ^1^Department of Health Psychology and Pedagogy, Rīgas Stradiņš University, Riga, Latvia; ^2^Faculty of Communication, Rīgas Stradiņš University, Riga, Latvia; ^3^Institute of Psychology, Faculty of Social Sciences, University of Tartu, Tartu, Estonia; ^4^Department of Neurology and Neurosurgery, Faculty of Medicine and Preventive Sciences, McGill University, Montreal, QC, Canada; ^5^Department of Psychology, Daugavpils University, Daugavpils, Latvia; ^6^Department of Psychiatry and Narcology, Institute of Public Health, Rīgas Stradiņš University, Riga, Latvia; ^7^International Centre for Education and Research in Neuropsychiatry (ICERN), Samara State Medical University, Samara, Russia; ^8^Department of Psychiatry, Narcology, Psychotherapy and Clinical Psychology, Samara State Medical University, Samara, Russia; ^9^3rd Department of Psychiatry, School of Medicine, Faculty of Health Sciences, Aristotle University of Thessaloniki, Thessaloniki, Greece; ^10^Mental Health Section, Research Institute, Panhellenic Medical Association, Thessaloniki, Greece; ^11^Department of Psychiatry and Narcology, Rīgas Stradiš University, Riga, Latvia

**Keywords:** COVID-19, preventive behavior, fear, trust in information sources, threat appraisals, conspiracy beliefs

## Abstract

**Background:** While COVID-19 has rapidly spread around the world, and vaccines are not widely available to the general population, the World Health Organization outlines preventive behavior as the most effective way to limit the rapid spread of the virus. Preventive behavior is associated with a number of factors that both encourage and discourage prevention.

**Aim:** The aim of this research was to study COVID-19 threat appraisal, fear of COVID-19, trust in COVID-19 information sources, COVID-19 conspiracy beliefs and the relationship of socio-demographic variables (gender, age, level of education, place of residence, and employment status) to COVID-19 preventive behavior.

**Methods:** The data originate from a national cross-sectional online survey (*N* = 2,608) undertaken in July 2020. The data were analyzed using structural equation modeling.

**Results:** COVID-19 threat appraisal, trust in COVID-19 information sources, and fear of COVID-19 are all significant predictors of COVID-19 preventive behaviors. Together they explain 26.7% of the variance of this variable. COVID-19 conspiracy beliefs significantly negatively predict COVID-19 threat appraisal (*R*^2^ = 0.206) and trust in COVID-19 information sources (*R*^2^ = 0.190). COVID-19 threat appraisal contributes significantly and directly to the explanation of the fear of COVID-19 (*R*^2^ = 0.134). Directly, as well as mediated by COVID-19 conspiracy beliefs, threat appraisal predicts trust in COVID-19 information sources (*R*^2^ = 0.190). The relationship between COVID-19 threat appraisal and COVID-19 preventive behaviors is partially mediated by fear of COVID-19 (indirect effect 28.6%) and trust in information sources (15.8%). Socio-demographic variables add very little in prediction of COVID-19 preventive behavior.

**Conclusions:** The study results demonstrate that COVID-19 threat appraisal is the most important factor associated with COVID-19 preventive behavior. Those Latvian residents with higher COVID-19 threat appraisal, experienced higher levels of fear of COVID-19, had more trust in COVID-19 information sources, and were more actively involved in following COVID-19 preventive behaviors. COVID-19 conspiracy beliefs negatively predict COVID-19 threat appraisal and trust in COVID-19 information sources, but not the COVID-19 preventive behaviors. Socio-demographic factors do not play an important role here.

## Introduction

As emphasized by the World Health Organization (World Health Organization, 2020) during the COVID-19 pandemic, and based on experience from previous twenty first century pandemics and virus outbreaks, preventive behavior is the most effective way to limit the spread of the virus while the vaccine is not available to the general public (Leppin and Aro, 2009; Rubin et al., 2009; Miao and Huang, 2012; World Health Organization, 2020).

Preventive behavior is studied within the framework of various theories of health behavior. This study integrates the Protection Motivation Theory (PMT), developed by Rogers (1975) and is still widely used in health psychology research (Miraja et al., 2019; Adunlin et al., 2020; Kowalski and Black, 2021). Preventive behavior can be defined as a combination of beliefs, attitudes and experience that motivate people to take actions in order to maintain and improve their prevention (Werle, 2011; Kowalski and Black, 2021; Rad et al., 2021). Aspects of preventive behavior such as social/physical distancing and observance of personal hygiene have become relevant in the conditions caused by the COVID-19 pandemic (Adunlin et al., 2020; Barati et al., 2020). Preventive behavior during a pandemic is essential not only for protection of individuals from being infected, but also for reduction of spread of the virus among the population, thus protecting vulnerable groups and society as a whole (Chuang et al., 2015; Kowalski and Black, 2021; Ranjit et al., 2021).

According to the PMT, preventive behavior is associated with threat assessment, which includes the assessment of the danger of the disease and its severity (Floyd et al., 2000; Barati et al., 2020). Studies have shown that optimal risk assessment promotes engagement in preventative behaviors to avoid disease, while an inadequate assessment of low risk can lead to non-compliance with recommended precautions, including preventive behavior (Ferrer and Klein, 2015; Miraja et al., 2019; Okuhara et al., 2020; Rad et al., 2021; Wang et al., 2021). In a cross-sectional study conducted in 10 countries during the COVID-19 pandemic, researchers found a statistically significant correlation between threat appraisal and preventive behavior (such as washing hands, wearing a face mask, and physical distancing) (Dryhurst et al., 2020). According to PMT, threat assessment is associated with fear (Miraja et al., 2019; Adunlin et al., 2020; Taheri-Kharameh et al., 2020; Rad et al., 2021).

Fear is defined as an unpleasant emotion that arises when an individual perceives threatening stimulus (de Hoog et al., 2008). According to PMT, fear is essential for a patient to change their behavior to avoid getting sick (Adunlin et al., 2020; Harper et al., 2020; Taheri-Kharameh et al., 2020) (Ahorsu et al., 2020; Chen et al., 2020; Pasion et al., 2020). Due to the rapid spread of the COVID-19 and its particular danger to certain vulnerable groups, fear and threat appraisal is an important factor that may contribute to an individual's involvement in preventive behavior to protect the relatives and significant others (Bitan et al., 2020; Jørgensen et al., 2020; Sahoo et al., 2020). Several studies have reported on positive correlations between fear of one's own and relatives' threat appraisals and preventive behavior (Balkhi et al., 2020; Parlapani et al., 2020; Sahoo et al., 2020). In the context of the COVID-19 pandemic, an excessive fear at the individual level can often lead to mental problems (Belen, 2020; Fountoulakis et al., 2020). However, a lack of fear may prevent individuals from participating in preventive measures to reduce the spread of COVID-19 (Gerritsenb, 2020; Taheri-Kharameh et al., 2020).

Information about potential threats to one's own or other people's health is an essential prerequisite for a change in behavior. For the first time in the history of all civilizations, society is experiencing a pandemic of this magnitude, resulting in a lack of both previous experience and evidence-based knowledge at the societal and individual levels (Azlan et al., 2020; Chesser et al., 2020). At the same time, information of very different content and quality is disseminated through various media and social channels. Studies carried out during the COVID-19 pandemic revealed a relationship between trust in information provided by the government, healthcare institutions, and news disseminated by mass media and preventive behavior (Al-Rasheed, 2020; Khosravi, 2020), as well as a negative relationship between belief in conspiracy theories and preventive behavior (Allington et al., 2020; Kim and Kim, 2021). Wang et al. (2021) in a study, conducted during the COVID-19 pandemic, highlights the relationship between different sources of information and risk perception and prevention behavior. Information, received from a variety of sources: healthcare professionals, colleagues, or collected on the Internet, is linked to a different threat appraisal, and threat appraisal is linked to the motivation to vaccinate. Consequently, the information sources and trust in specific information sources are important for risk perception and preventive behavior. Trust can be defined as an expectancy held by an individual or a group that the word, promise, verbal or written statement of another individual or group can be relied on (Al-Rasheed, 2020). A study, conducted during the COVID-19 pandemic, showed a strong positive correlation between trust in the government and preventive behavior (Al-Rasheed, 2020; Borgonovi and Pokropek, 2020; Khosravi, 2020) indicating that members of the society who have confidence that the information provided by the government and the recommended security measures are reliable and reasonable will comply with the security measures. The relationship between trust, threat perception, evaluation, and behavior is shown in the Trust Confidence and Cooperation Model (TCC), which was developed in order to explore trust and risk management and mutual collective collaboration (Siegrist et al., 2003). Therefore, during the COVID-19 pandemic, getting information from public health professionals, the government, and the news media can increase people's awareness of the risk, and consequently, their adoption of preventive behaviors (Siegrist et al., 2003; Bäuerle et al., 2020; Gopichandran et al., 2020; Khosravi, 2020; Siegrist, 2021). Similarly, research carried out during the COVID-19 pandemic suggests that information provided by the government, the healthcare system or media about the origin of the virus and its dangers could also create fears, which in turn can be a stimulus for behavioral changes (Cauberghe et al., 2009; Shirahmadi et al., 2020). Respectively, evidence-based information that appeals to fear and is trusted by the public motivates involvement in measures taken to control the spread of the virus.

Belief in conspiracy theories refers to preventive beliefs on suspicions of covert and malicious actions by government, institutions or organizations. Circumstances, where information about a topical issue is incomplete, or there is too much information and this information is negative (Marchlewska et al., 2018), provide particularly favorable conditions for the spread of conspiracy theories. With the worldwide spread of COVID-19, conspiracy theories have developed and spread rapidly, offering a variety of explanations for the causes of the virus and its purposes (Gogarty and Hagle, 2020). In this case, conspiracy theories provide a broad, internally coherent, but objectively unverifiable explanation, creating a false sense of internal security in an environment of external insecurity and uncertainty (Douglas et al., 2017). The recent literature shows that belief in conspiracy theories can affect a realistic threat assessment as well as undermine confidence in evidence-based, science-based information (Banai et al., 2020; Sobkow et al., 2020; Heiss et al., 2021) thus influencing the individual's threat appraisal and involvement in the preventive behavior (Allington et al., 2020; Heiss et al., 2021; Kim and Kim, 2021; Ranjit et al., 2021).

Previous research has demonstrated that demographic differences (e.g., female and more educated) are significantly associated with engagement in protective behaviors (Floyd et al., 2000; Cvetković et al., 2020; Dohle et al., 2020; Rad et al., 2021; Yildirim et al., 2021). Regarding the differences in fears and threat assessments across socio-demographic groups, several researchers argue that younger people experience higher threat assessments and fears, but getting older threat assessment and fear decrease (Russac et al., 2007; Pasion et al., 2020; Yildirim et al., 2021), however, other studies, carried out during the COVID-19 pandemic, show that women and older people in particular are more concerned about COVID-19 and the health risks (Miraja et al., 2019; Adunlin et al., 2020; Hossain et al., 2020). Respectively, during the COVID-19 pandemic women and older people appreciate the seriousness of the situation, the danger of the disease, and fear of COVID-19 (Barati et al., 2020; Okuhara et al., 2020; Rad et al., 2021). Researchers have received different results regarding trust in information sources (Al-Rasheed, 2020; Khosravi, 2020). For example, as to the information provided by scientists on the safety of vaccines, the results of the study show that women show lower confidence (Latkin et al., 2021), while another study found that it was women who had higher confidence in evidence-based information (Algara et al., 2020; Latkin et al., 2021). One more study finds that younger people with higher education have more confidence in evidence-based information, but there is no gender difference (Borgonovi and Pokropek, 2020). As for belief in various conspiracy theories, part of the research conducted during the COVID-19 pandemic found no differences between age, gender and level of education in relation to COVID-19 conspiracy beliefs (Pasion et al., 2020; Pummerer et al., 2021). Another study showed differences in socio-demographic factors, in particular younger women with lower levels of education were more likely to believe in conspiracy theories (Pickles et al., 2020).

Basing on an extensive literature review, we have identified factors that are important for the implementation of preventive behavior to reduce the prevalence of COVID-19. As part of this study, a combined model has been described in which we have included elements of PMT: fear, threat assessment, and the relationship of these elements with health behavior. Based on the TCC, we have examined the relationship between trust in COVID-19 information sources and threat assessment and COVID-19 preventive behavior (as involvement in collective action) and the relationship between belief conspiracy theories and socio-demographic factors and the elements included in the model.

The aim of this study was to discover the relationship between COVID-19 threat assessment, fear of COVID-19, trust in COVID-19 information sources, conspiracy theories and socio-demographic factors (gender, age, level of education, place of residence and employment status) and COVID-19 preventive behavior.

The following hypotheses were formulated based on the aforementioned literature:

H1: Belief in COVID-19 conspiracy theories will be negatively associated with trust in COVID-19 information sources.
H2: Trust in COVID-19 information sources will be positively associated with fear of COVID-19.
H3: Belief in COVID-19 conspiracy theories will be negatively associated with COVID-19 risk appraisal.
H4: Trust in COVID-19 information sources will be positively associated with COVID-19 risk appraisal.
H5: Fear of COVID-19 will be positively associated with COVID-19 risk appraisal.
H6: Belief in COVID-19 conspiracy theories will be negatively associated with COVID-19 preventive behaviors.
H7: COVID-19 Risk appraisal will be positively associated with COVID-19 preventive behaviors.
H8: Fear of COVID-19 will be positively associated with COVID-19 preventive behaviors.
H9: Trust in COVID-19 information sources will be positively associated with COVID-19 preventive behaviors.
H10: There is no association between socio-demographic variables and trust in COVID-19 information sources.
H11: Women and older people will have higher rates of fear of COVID-19 and threat appraisal.
H12: Women and older people will more frequently engage in COVID-19 preventive behaviors.
H13: Relationship between fear of COVID-19 and engagement in COVID-19 preventive behaviors will be at least partially mediated by COVID-19 risk appraisal.
H14: Relationship between trust in COVID-19 information sources and engagement in COVID-19 preventive behaviors will be at least partially mediated by COVID-19 risk appraisal.

## Materials and Methods

A cross-sectional national online survey was conducted in Latvia to examine the association of COVID-19 preventive behaviors with trust in COVID-19 information sources regarding pandemic control, COVID-19 threat appraisal, COVID-19 conspiracy beliefs, and fear of COVID-19. The information sources included the government, news media, and the health care system.

### The Survey

A quantitative cross-sectional online survey was carried out with a sample of the Latvian general population aged 18–74 years in the frame of the Latvian National Research Program (No. VPP-COVID-2020/1-0011) and in collaboration with the Mental Health Sector of the Scientific Research Institute of the Pan-Hellenic Medical Association. The full survey consisted of 27 thematic sections, including socio-demographic questions (gender, age, education, living place location, and employment status), and sections with questions about conspiracy beliefs, fear of COVID-19, COVID-19 threat appraisal, COVID-19 prevention measures practiced, and trust in information sources. The questionnaire was available in Latvian and Russian languages, and both versions of the questionnaire were studied by Latvian and Russian speaking focus groups in order to adapt them before distribution. The first half of the survey, including parts about conspiracy theories and thoughts and fears about COVID-19, consisted of questions used in the international survey entitled “Estimating the Effects of COVID-19 Outbreak on Mental Health (Fountoulakis et al., 2020; Patsali et al., 2020).”

### Data Collection Procedure

The study was conducted as an online survey from July 6th to July 27th, 2020. A carefully selected and segmented database corresponding to the general population of Latvia was used. Respondents received individual invitations by e-mail, with a password and a link to an online questionnaire, which could be completed by respondents at their preferred time until the specified survey closing time. There were two options for the language of instructions offered to participants— Latvian or Russian. To ensure the security of data transmission, the SSL (Secure Sockets Layer) data transmission protocol was used. Reminders about filling in the questionnaire were sent to respondents' e-mails. When the respondent filled out the questionnaire, it was saved on KANTAR's server and was not available for later editing. Data processing and analysis were carried out after the survey was closed. Only fully completed questionnaires were included in the database.

### Participants

The total sample size was 2,608, but 2,606 participants were included in the analysis, because the questionnaires completed by two participants were found to be invalid. A total of 1,036 (39.8%) male, and 1,570 (60.2%) female participants completed the survey. They all were residents of Latvia, aged 18–75 (*M* = 46.42, *SD* = 13.86). More precisely, 6.4% of the participants were aged between 18 and 25 years, 20.4% were aged between 26 and 35 years, 19.1% were between 36 and 45 years, 26.9% between 46 and 55 years, 18.2% between 56 and 65 years, and 9.0% were older than 66 years. Most had completed higher secondary education (12 years or equivalent level of education) (36.9%), 29.8% had a bachelor's degree, 29.4% had a master's degree, 1.5% had a PhD or an equivalent level of education and 2.4% had a general primary education (9 years of education). The majority of the sample (73.2%) currently live in an urban area (53.0% of them in the capital city of Latvia), and were employed (71.8%). More than two thirds (68.0%) completed the survey in Latvian, and 32.0% in Russian.

### Variables

#### COVID-19 Preventive Behavior

COVID-19 preventive behavior was measured using a subset of seven items, selected basing on the item content from the survey part labeled “Changes in the behavior of the population as a result of the COVID-19 pandemic”: two items regarding compliance with hygiene recommendations (No. 1: “*I started washing my hands more frequently and thoroughly*” and No. 3: “*I started using disinfectants regularly every day*”) and four items regarding social distancing (No. 10: “*I avoid leaving home if not necessary*,” No. 12: “*I tend to stay less frequently in public places*,” No. 13: “*I try to avoid direct contact with other people*,” No. 14: “*I try to avoid contact with people not belonging to my household (as often as possible*),” No. 15: “*I try to maintain social distance in public places*”). In the introductory part for these items, participants received the following instructions: “*During the state of emergency, the government imposed a number of restrictions aimed at reducing the spread of COVID-19. We are interested in how your behavior has changed since the announcement of the state of emergency, compared to the time before the state of emergency*.” All items in this part of the survey were answered on a response scale from 1 to 5 (“Disagree” to “Agree”) and were originally formulated for this survey. The scale exhibited good internal consistency with Cronbach's α = 0.87 in the total sample, α = 0.87 for the Latvian version, and α = 0.88 for the Russian version. An average score was computed to create a composite variable for further analysis.

#### Trust in COVID-19 Information Sources

To evaluate the trust in COVID-19 information sources, respondents were asked: “*Please assess the extent to which you personally trust each of the institutions listed below regarding the provided information and behavior recommendations during the state of emergency:* (1) *Government*, (2) *News media*, (3) *Health care system*.” The response scale ranges from 1 (“*I do not trust this institution at all*”) to 10 (“*I fully trust this institution*”). As the three items were reasonably highly correlated (*r* = 0.55–0.66, *p* < 0.001), they were treated as indicators of trust in COVID-19 information sources. The scale exhibited good internal consistency in the total sample (α = 0.83), and for the Latvian (α = 0.83) and Russian versions (α = 0.81). An average score was computed to create a composite variable for further analysis.

#### Fear of COVID-19

To evaluate the fear of COVID-19 respondents were asked the following questions: “*Are you afraid that you will contract the coronavirus?*” and “*Does the possibility that a member of your family could contract the coronavirus and die because of it, make you frightened?*” The response scale ranges from 1 to 5 (“Never” to “Very Much”). Because the two questions were highly correlated (*r* = 0.60, *p* < 0.001), they were treated as indicators of the fear of COVID-19. The scale exhibited good internal consistency in the total sample (α = 0.74), for the Latvian version (α = 0.73), and for the Russian version (α = 0.77). An average score was computed to create a composite variable for further analysis.

#### COVID-19 Conspiracy Beliefs

To evaluate the COVID-19 conspiracy beliefs, respondents were asked the following questions: “*Do you believe that COVID-19 was created in a laboratory to be used as a biochemical weapon for the extermination of the human population?*” and “*Do you believe that COVID-19 is a creation of the world's powerful leaders to create a global economic crisis?*” The response scale ranged from 1 to 5 (“I don't believe it at all” to “Very much”). As the two questions were highly correlated (*r* = 0.65, *p* < 0.001) and we were interested in general conspiracy beliefs about COVID-19, these two items were treated as indicators of the COVID-19 conspiracy beliefs. The scale had good internal consistency in the total sample (α = 0.79)—for the Latvian version (α = 0.81), and for the Russian version (α = 0.76). An average score was calculated to yield a composite variable for further analysis. Questions to assess fear of COVID-19 and COVID-19 conspiracy beliefs were taken from the Mental Health Sector Survey of the Scientific Research Institute of the Pan-Hellenic Medical Association “Assessment of the Impact of the COVID-19 Outbreak on Mental Health”.

#### COVID-19 Threat Appraisal

To evaluate the COVID-19 threat appraisal, the respondents were asked: “*Please assess to what extent you agree with the following statements about COVID-19:* (1) *The danger of this virus is greatly exaggerated;* (2) *I am convinced that the situation is not as serious as it is reported by the mass media*.” The response scale ranged from 1 to 5 (“Disagree” to “Agree”). Both questions were originally formulated for this survey. A reverse coding was used for both questions so higher scores represent higher threat appraisal. Both questions are highly correlated (*r* = 0.78, *p* < 0.001), so were treated as indicators of the COVID-19 threat appraisal. The scale exhibited good internal consistency in the total sample (α = 0.88), for the Latvian version (α = 0.87), and for the Russian version (α = 0.88). An average score was computed to create a composite variable used for further analysis.

### Covariates

The following socio-demographic data were collected during the study and were evaluated as covariates when performing the structural equation modeling (SEM) analysis: age, gender (0 = “female”, 1 = “male”), education level (0 = “secondary or lower”) i.e., combination of such levels as: “basic education (5 years of school) or lower”, “compulsory education (9 years of school),” “secondary/professional education (12 years of school)”; 1 = “higher education” (i.e., combination of levels such as: Bachelor's degree, Master's degree, and PhD); living place location (0 = “urban” i.e., categories like: “capital city,” “city >1 million population,” “city (100.000–1 million population),” “town (20,000–100,000 inhabitants),” “town (<20.000 inhabitants)”; 1 = “rural” i.e., response category: “rural area—village”), and employment status (0 = “unemployed” i.e., a combination of categories such as: “pensioner,” “unemployed,” “housewife,” “pension due to health,” “college or university student”; 1 = “employed” i.e., categories such as: “work in the public sector,” “employee in the private sector,” “self-employed/freelancer”).

### Data Analysis

The sample characteristics were described using frequencies and means of age, gender, education level, living place location and employment status. Descriptive statistics for the main variables and correlations between them were obtained. Cronbach's alpha was calculated to estimate the reliability of each scale (composite variable). In the following analyses we used SEM—a confirmatory approach of model validation. All items of the reported instruments were used as indicators of the respective latent variable in the SEM, and a few pairs of items with similar content (within the same scale) were allowed correlated measurement errors.

First, confirmatory factor analysis (CFA) was used to test the proposed measurement models of the latent variables (i.e., to verify the “fit” of the observed variables for each latent variable). Then, structural models were examined to assess the relationships between the variables. For each model tested we assessed overall fit (Kline, 2005) the significance of individual structural paths (Hu and Bentler, 1999) and the amount of variability (Douglas et al., 2017) R^2^ of the latent variables accounted for by observed variables. Model fit was assessed using the goodness-of-fit indices including the chi-square (χ2), Comparative Fit Index (CFI ≥ 0.90 is acceptable, ≥0.95 is good) (Kenny, 2020), Root Mean Square Error of Approximation (RMSEA ≤ 0.08 recommended) and Standardized Root Mean Residual (SRMR ≤ 0.08 recommended (Hu and Bentler, 1999; Kline, 2016). The CFI compares the existing model fit for a null model assuming uncorrelated variables (independence model). The RMSEA assesses overall fit but penalizes for less parsimonious models. The SRMR is an absolute measure of fit and is defined as the standardized difference between the observed and predicted correlations. Since the SRMR is an absolute measure of fit, a value of zero indicates perfect fit. The SRMR has no penalty for model complexity (Kenny, 2020).

**Figure 1:**
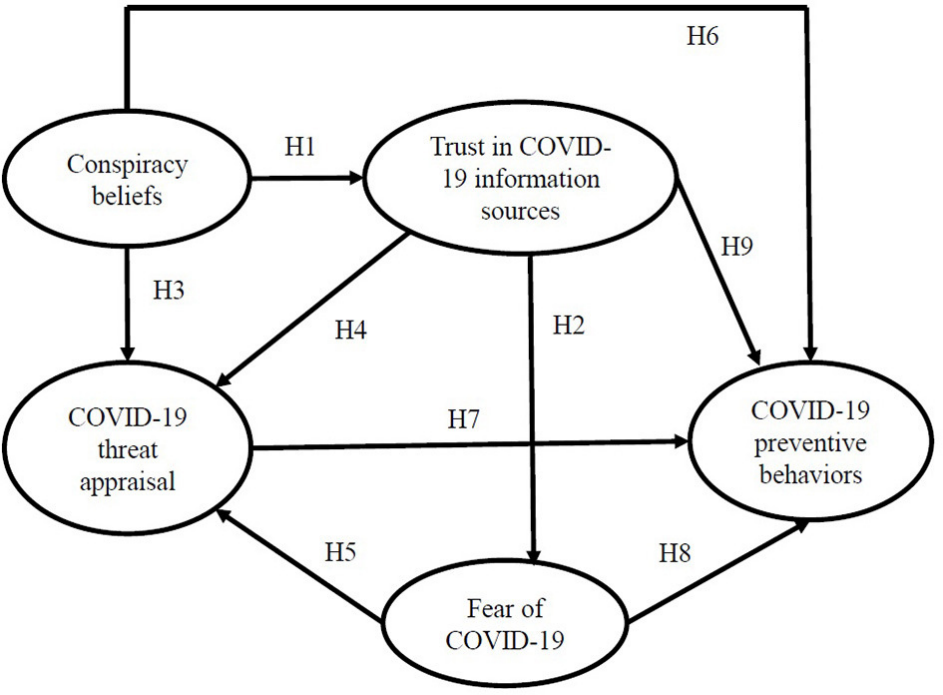
Theoretical model.

The two models (M1—the theoretical model as shown in Figure 1, and M2—the adapted model with an added full range of socio-demographic covariates) were compared using Akaike's Information Criterion (AIC) and Bayes Information Criterion (BIC). Lower values indicate a better fit, and so the model with the lowest AIC and BIC is the best fitting model. Standardized estimates for path coefficients, interpreted as regression coefficients, were calculated for all proposed relationships in the final model, as well as the relevant indirect effects to test the mediation hypotheses. As some variables were ordinal and not normally distributed, we used the robust maximum likelihood estimator (MLR) throughout the analyses. Modification indices were examined to improve the fit of the model according to theory and evidence from the correlation matrix (Kline, 2005). All analyses were performed using R 4.02. software. CFA and SEM analysis was performed using the lavaan package (Rosseel, 2012).

## Results

### Preliminary Analysis

We calculated correlations between all main variables at sum-score level (Table 1). As shown in the table, COVID-19 threat appraisals negatively correlated with COVID-19 conspiracy beliefs, and positively correlated with all the other variables. Similar patterns can be seen in the case of fear of COVID-19 and trust in COVID-19 information sources (total, and for each particular information source). COVID-19 conspiracy beliefs score is negatively correlated with all of the other variables, while the correlation coefficient related to fear of COVID-19 is very weak in magnitude.COVID-19 preventive behaviors are most strongly correlated with COVID-19 threat appraisal and fear of COVID-19 (both medium in magnitude); it weakly correlated with total score of trust in COVID-19 information sources and with trust in each of three separate COVID-19 information sources, and negatively (weak in magnitude) correlated with COVID-19 conspiracy beliefs.

**Table 1:** Descriptive statistics and correlations between variables.

**Variable**	***M***	***SD***	**1**	**2**	**3**	**4**	**5**	**5.1**	**5.2**
1. COVID-19 preventive behaviors	3.70	1.01	–						
2. COVID-19 conspiracy beliefs	2.50	1.14	−0.17^***^	–					
3. COVID-19 threat appraisal	3.12	1.25	0.40^***^	−0.45^***^	–				
4. Fear of COVID-19	2.36	0.94	0.42^***^	−0.06^**^	0.37^***^	–			
5. Trust in COVID-19 information sources	4.99	2.13	0.29^***^	−0.44^***^	0.45^***^	0.14^***^	–		
5.1. Trust in government	4.49	2.70	0.26^***^	−0.43^***^	0.41^***^	0.11^***^	0.89^***^	–	
5.2. Trust in mass media	4.77	2.22	0.25^***^	−0.34^***^	0.42^***^	0.13^***^	0.84^***^	0.63^***^	–
5.3. Trust in health system	5.69	2.4	0.25^***^	−0.36^***^	0.34^***^	0.11^***^	0.87^***^	0.66^***^	0.59^***^

** *p < 0.010*,

*** *p < 0.001*.

*N = 2,606. For all variables scores can range from 1 to 5*.

To verify whether the sum-scores are appropriate, we replicated the associations using SEM modeling. Instead of sum-scores we used latent variables. After small adjustments (i.e., allowing correlated measurement errors based on modification indices between two pairs of items within the preventive behavior scale: No. 3 (“*I started using disinfectants regularly every day*”); No. 3 (“*I started washing my hands more frequently and thoroughly*”); No. 13 (“*I try to avoid direct contact with other people*”), and No. 14 (“*I try to meet people who do not belong to my household as rarely as possible”*), CFA showed acceptable to good model fit for all latent variables (COVID-19 conspiracy beliefs, COVID-19 threat appraisal, trust in COVID-19 information sources, fear of COVID-19 and COVID-19 preventive behaviors). The final model fit was very good {Robust CFI = 0.98, Robust RMSEA = 0.042 [90% CI (0.038, 0.046)], SRMR = 0.036}. The correlations between latent variables were similar to the ones reported in Table 1. This analysis suggests that the sum-scores used are good approximations of the data. We preferred sum-scores to latent variables for variables in the analyses below for the sake of simplicity.

### Model Testing

First, we tested the baseline model (Model 1), which contained the first five variables presented in Table 1 and all possible links between them (without links between conspiracy beliefs and fear due to too low correlation coefficient between these two variables). The fit of this model is displayed in Table 2.

**Table 2:** Model fit indices for SEM of COVID-19 preventive behaviors.

**SEM model**	**χ^2^**			**CFI**	**RMSEA**	**90% confidence interval**		**SRMR**	**AIC**	**BIC**
	**χ^2^**	**(df)**	***p***			**Lower bound**	**Upper bound**			
Model 1	0.01	1	=0.912	1.00	0.000	0.001	0.021	0.001	31816.16	31892.41
Model 2	0.04	1	=0.850	1.00	0.000	0.000	0.029	0.001	31629.33	31822.90

^***^p < 0.001. CFI, comparative fit index (CFI ≥ 0.90 is acceptable, ≥0.95 is good); RMSEA, root mean square error of approximation (RMSEA ≤ 0.08 recommended); SRMR, standardized root mean residual (SRMR ≤ 0.08 recommended); AIC, Akaike's information criterion; BIC, Bayes information criterion (BIC) (lower values indicate a better fit, therefore the model with the lowest AIC and BIC is the best fitting model). Model 1, the baseline model which contained first five variables presented in Table 1 and all possible links between them (without a link between conspiracy beliefs and fear due to too low correlation coefficient between these two variables). Model 2, a more complicated version of Model 1 with the socio-demographic variables added as covariates.

**Figure 2:**
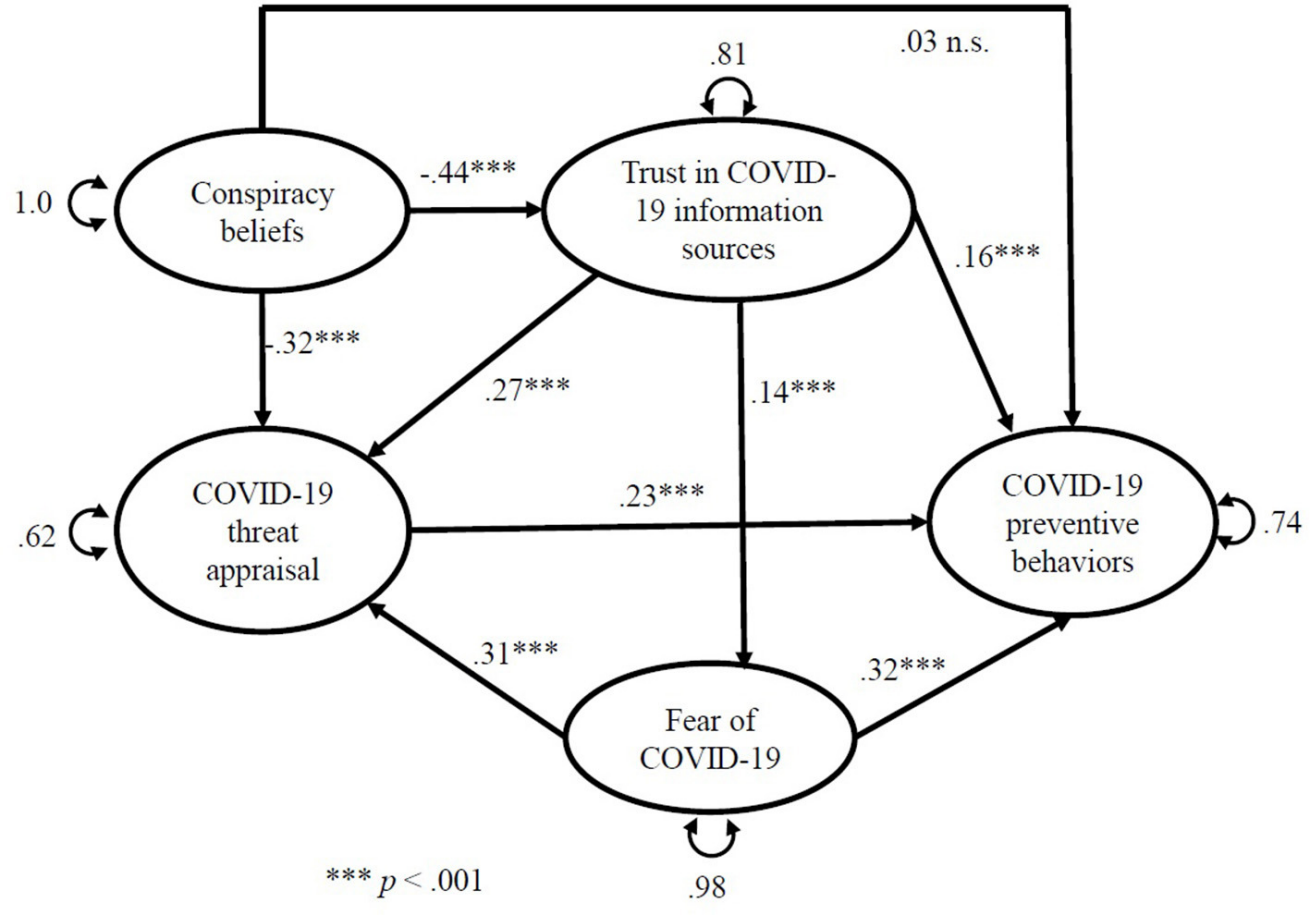
Structural equation model of Model 1.

The estimates of each structural relationship between the Model 1 variables are shown in Figure 2 and Table 3. COVID-19 conspiracy beliefs negatively predict trust in COVID-19 information sources (*R*^2^= 0.190). Trust in COVID-19 information sources significantly positively predicts fear of COVID-19 (*R*^2^ = 0.019). COVID-19 conspiracy beliefs significantly negatively, but trust in COVID-19 information sources and fear of COVID-19 positively predict COVID-19 threat appraisal (all together they explain 37.8% of the variance of this variable). COVID-19 threat appraisal, trust in COVID-19 information sources and fear of COVID-19 are all significant predictors of COVID-19 preventive behaviors. Together they explain 26.5% of the variance of this variable. Path between COVID-19 conspiracy beliefs and COVID-19 preventive behaviors is not statistically significant (ß = 0.03, *p* =0.22) (see Table 3).

**Table 3:** Standardized path coefficients predicting COVID-19 preventive behaviors (Model 1).

**Structural path**	**Coefficient**	**SE**	***Z***	***p* > |*z*|**	**ß**	***R^**2**^***
Trust in information sources						0.190
Conspiracy beliefs	−0.81	0.03	−24.76	<0.001^***^	−0.44	
Fear						0.019
Trust in information sources	0.06	0.01	7.04	<0.001^***^	0.14	
Threat appraisal						0.378
Conspiracy beliefs	−0.35	0.02	−18.61	<0.001^***^	−0.32	
Trust in information sources	0.16	0.01	15.38	<0.001^***^	0.27	
Fear	0.41	0.02	19.96	<0.001^***^	0.31	
Preventive behavior						0.265
Threat appraisal	0.18	0.02	10.59	<0.001^***^	0.23	
Trust in information sources	0.08	0.01	8.11	<0.001^***^	0.16	
Fear	0.34	0.02	17.33	<0.001^***^	0.32	
Conspiracy beliefs	0.02	0.02	1.24	0.22	0.03	

We also tested a more complicated model with the socio-demographic variables added as covariates (Model 2). This model exhibited a slightly better fit, as expressed by the AIC and BIC values and other fit indices (see Table 2), but based on R^2^ change, these variables add very little in prediction of dependent variables. As Table 4 shows, living in a rural area, possessing higher education, and being employed were significantly related to trust in COVID-19 information sources, but these variables added only 0.9% to the explained variance of this dependent variable. Next, it was found that age (being younger), gender (being female), and possessing higher education is significantly related to fear of COVID-19, but incremental value of these variables is only 1.3%. For COVID-19 threat appraisal, age (being older), and education (high education levels) are significant predictors of this variable. However, in sum, socio-demographic variables add only 1.0% in the explanation of COVID-19 threat appraisal variance. Finally, in the prediction of COVID-19 preventive behaviors only age (being older) and gender (being female)—but not education, living place location and employment status—are significant predictors along with the threat appraisal, trust in information sources and fear of COVID-19. In this case, demographic variables add 3.3% of explained variance of the preventive behavior score.

**Table 4:** Standardized path coefficients predicting COVID-19 preventive behaviors (Model 2).

**Structural path**	**Coefficient**	**SE**	***Z***	***p* > |*z*|**	**ß**	***R^**2**^***
Trust in information sources						0.199
Conspiracy beliefs	−0.81	0.03	−24.76	<0.001^***^	−0.44	
Age	−0.00	0.00	−0.75	0.451	−0.01	
Gender	−0.10	0.08	−1.23	0.219	−0.02	
Education	0.27	0.08	3.43	0.001^**^	0.06	
Location	0.29	0.09	3.38	0.001^**^	0.06	
Employment	−0.17	0.09	−1.99	<0.046^*^	−0.04	
Fear						0.032
Trust in information sources	0.06	0.01	6.87	<0.001^***^	0.13	
Age	−0.00	0.00	−2.52	0.012^*^	−0.05	
Gender	−0.18	0.04	−4.82	<0.001^***^	−0.10	
Education	0.05	0.04	1.30	0.019^*^	0.03	
Location	−0.02	0.04	−0.43	0.668	−0.01	
Employment	−0.02	0.04	−0.49	0.624	−0.01	
Threat appraisal						0.388
Conspiracy beliefs	−0.35	0.02	−18.31	<0.001^***^	−0.32	
Trust in information sources	0.16	0.01	15.21	<0.001^***^	0.27	
Fear	0.42	0.02	20.43	<0.001^***^	0.32	
Age	0.01	0.00	5.33	<0.001^***^	0.08	
Gender	0.07	0.04	1.77	0.078	0.03	
Education	0.10	0.04	2.35	0.019^*^	0.04	
Location	0.05	0.04	1.21	0.228	0.02	
Employment	−0.01	0.04	−0.018	0.854	−0.00	
Preventive behavior						0.298
Threat appraisal	0.17	0.02	10.09	<0.001^***^	0.21	
Trust in information sources	0.08	0.01	8.13	<0.001^***^	0.16	
Fear	0.34	0.02	17.39	<0.001^***^	0.31	
Conspiracy beliefs	0.01	0.02	0.39	00.70	0.01	
Age	0.01	0.00	7.69	<0.001^***^	0.13	
Gender	−0.28	0.04	−7.92	<0.001^***^	−0.13	
Education	0.06	0.04	1.79	0.072	0.03	
Location	−0.00	0.04	−0.06	0.949	−0.00	
Employment	0.02	0.04	0.46	0.642	0.01	

* *p < 0.05*,

** *p < 0.01*,

*** *p < 0.001*.

*Gender: 0, “female”; 1, “male”. Education: 0, “secondary or lower”; 1, “higher education”. Living place location: 0, “urban”; 1, “rural”. Employment: 0, “unemployed”; 1, “employed”*.

We also investigated the mediating effect of COVID-19 threat appraisals, in the relationship between both fear of COVID-19 and trust in COVID-19 information sources as independent variables and COVID-19 preventive behaviors as the dependent variable. We estimated indirect effects, presented in Table 5. The results suggest that fear of COVID-19 and trust in COVID-19 information sources exert not only a direct effect, but also an indirect effect on COVID-19 preventive behaviors via COVID-19 threat appraisals (which mediated 25.1% in the first case, and 51.7% in the second case, based on proportion: indirect effect/total effect) (Table 5).

**Table 5:** Estimation of indirect and total effects.

**Mediation model**	**Effect type**	**Parameter estimates**					
		**Unstan-dardized**	**S.E**.	***p*-value**	**Confidence Interval**		**Stan-dardized**
					**Lower**	**Upper**	
Fear → Threat	Indirect	0.113	0.010	<0.001^***^	0.094	0.131	0.105
appraisal → Preventive	Total	0.450	0.020	<0.001^***^	0.408	0.491	0.418
behavior	Proportion	0.251	0.022	<0.001^***^	0.207	0.292	0.251
Trust in information	Indirect	0.072	0.005	<0.001^***^	0.062	0.083	0.151
sources → Threat	Total	0.139	0.010	<0.001^***^	0.121	0.158	0.293
appraisal → Preventive behavior	Proportion	0.517	0.049	<0.001^***^	0.432	0.622	0.517

*** p < 0.001. Indirect effect = (a*b) = (c – c′). Total effect = [c' + (a*b)]. Proportion = (indirect/total).

**Figure 3:**
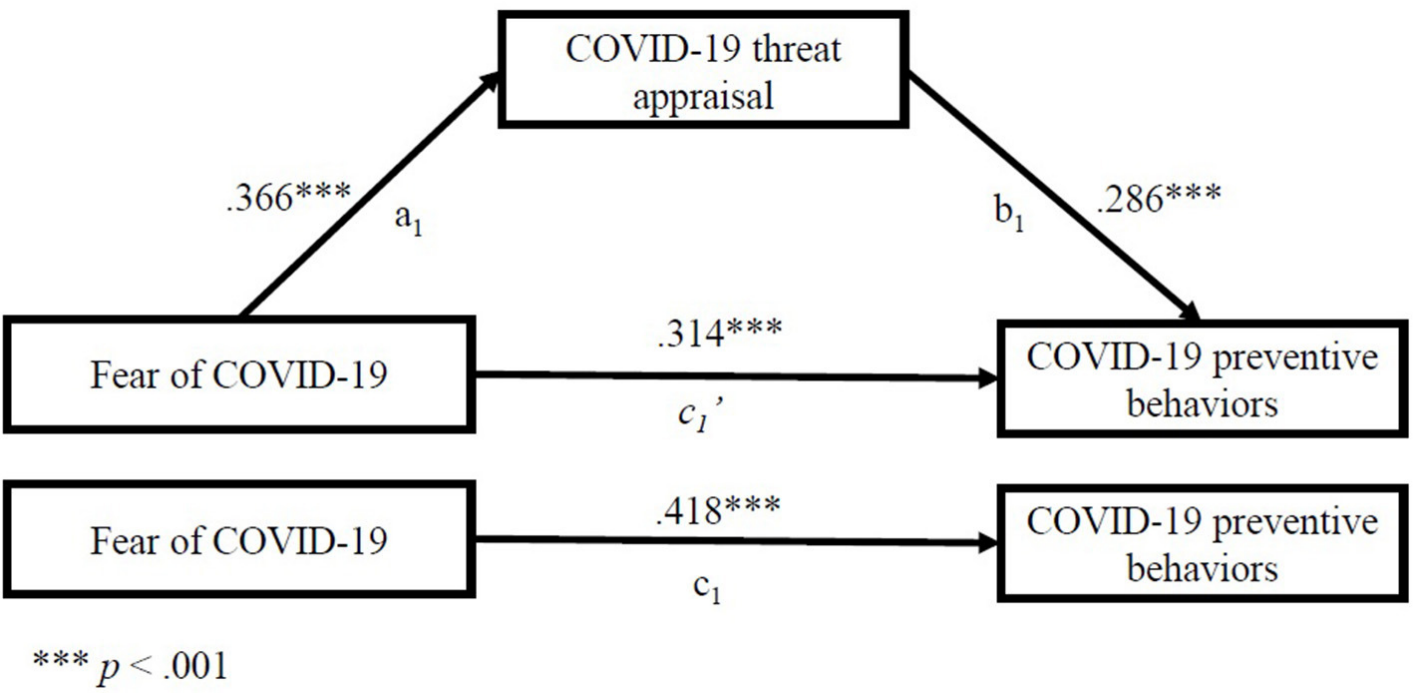
Standardized regression coefficients for the relationship between fear of COVID-19 and COVID-19 preventive behaviors as mediated by COVID-19 threat appraisal.

As Figure 3 illustrates, the standardized regression coefficient between fear of COVID-19 and a mediator—COVID-19 threat appraisal (a_1_ path) was statistically significant, as was the standardized regression coefficient between the mediator and dependent variable—COVID-19 preventive behaviors (b_1_). The standardized indirect effect (a_1_b_1_) was (0.366) x (0.286) = 0.105 (*p* < 0.001). It was also found that fear of COVID-19 was associated with the COVID-19 preventive behavior score, also independently of its association with COVID-19 threat appraisal, *p* < 0.001, so partial mediation was approved (prop = indirect effect/total effect = 0.251, *p* < 0.001).

**Figure 4:**
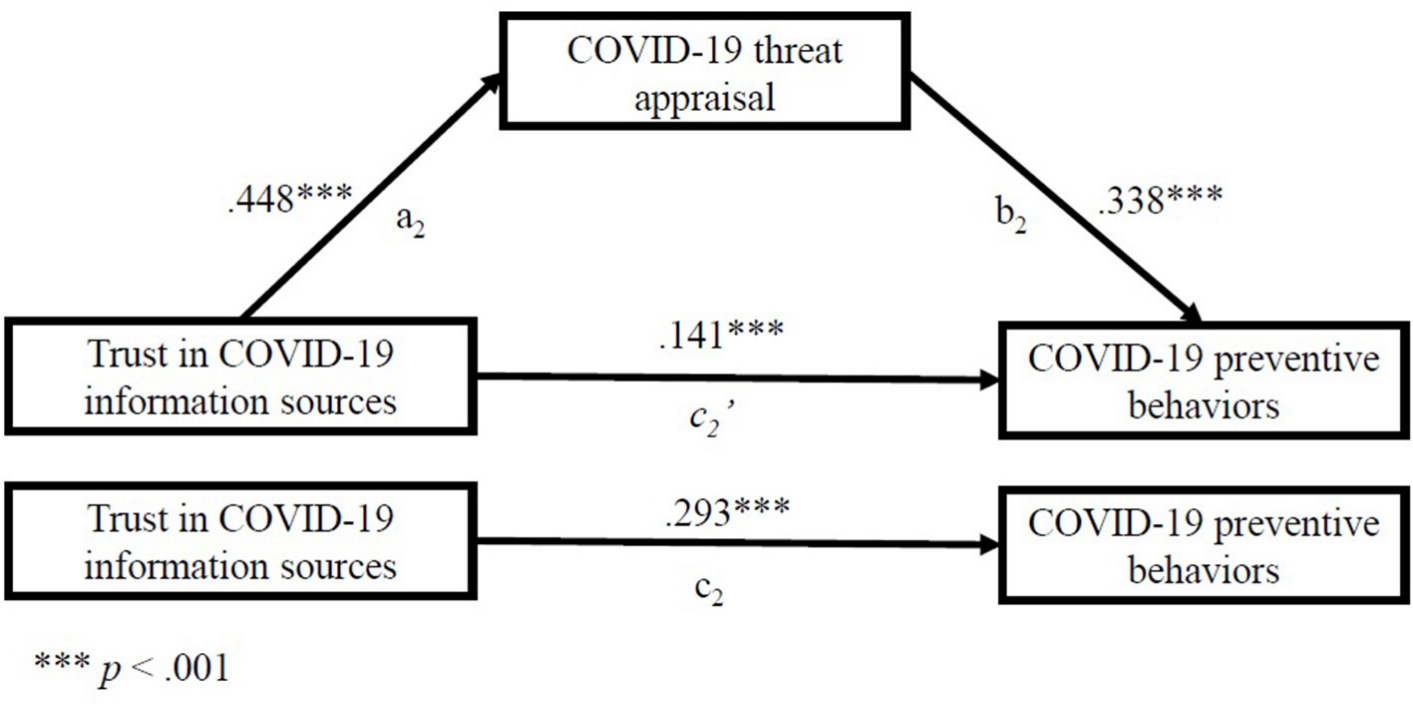
Standardized regression coefficients for the relationship between trust in COVID-19 information sources and COVID-19 preventive behaviors as mediated by COVID-19 threat appraisal.

As Figure 4 illustrates, the standardized regression coefficient between trust in COVID-19 information sources and a mediator—COVID-19 threat appraisal (a_2_ path) was statistically significant, as was the standardized regression coefficient between the mediator and dependent variable—COVID-19 preventive behaviors (b_2_). The standardized indirect effect (a_2_b_2_) was (0.448) x (0.338) = 0.151 (*p* < 0.001). It was also found that trust in COVID-19 information sources was associated with the COVID-19 preventive behavior score, also independently of its association with COVID-19 threat appraisal, *p* < 0.001, so partial mediation was approved (prop = indirect effect/total effect = 0.517, *p* < 0.001).

We tested the significance of these indirect effects using bootstrapping procedures. Unstandardized indirect effects were computed for each of 1,000 bootstrapped samples, and the 95% confidence interval was computed by determining the indirect effects at the 2.5 and 97.5th percentiles. The bootstrapped unstandardized indirect effect (a_1_b_1_) in the first mediation model was 0.011 (S.E. = 0.010), 95% CI (0.094, 0.131), and in the second mediation model the bootstrapped unstandardized indirect effect (a_2_b_2_) was 0.072 (S.E. = 0.005), 95% CI (0.062, 0.083). A bias-corrected bootstrapped confidence interval with 1,000 samples was above zero. Thus, the indirect effect in both cases was statistically significant (*p* < 0.001).

## Discussion

In this study, based on the PMT and TCC models, a combined model was described including variables such as COVID-19 preventive behavior, COVID-19 threat appraisal, COVID-19 conspiracy beliefs, fear of COVID-19, trust in COVID-19 information sources.

In order to test the interrelationships of the PMT factors included in the combined model and the relationship of these factors with the socio-demographic indicators, hypotheses nos. 5, 7, 8, 11, 12, 13 were formulated within this study. The results show that fear of COVID-19 is positively related to threat assessment (H5 supported), which confirms the mechanism explained by PMT. Respectively, fear as a strong emotional response is associated with cognitive assessment of potential health risk (Miraja et al., 2019; Adunlin et al., 2020; Van Bavel et al., 2020). The results also show that threat appraisal is closely associated with preventive behaviors (H7 supported), similar to the findings mentioned in other studies (Al-Rasheed, 2020; Wong et al., 2020; Kowalski and Black, 2021; Rad et al., 2021). According to the PMT, the higher is the perception of risk of infection, the greater is the likelihood that specific actions will be taken to avoid illness (Adunlin et al., 2020; Barati et al., 2020). According to data from Center for Disease Prevention and Control of Latvia, on 31.07.2020, the 14-day cumulative number of COVID-19 cases per 100 000 people was 2.6, and the total number of COVID-19 deaths, since the start of pandemic, was 32^1^. Thus, the results of this study show that even with a relatively small number of COVID-19 cases^2^ and fairly low potential of infection at the time of data collection, the threat appraisal of Latvian population regarding the possibility of being infected was at a sufficiently optimal level to motivate the implementation of preventive behavior. Looking at the relationship between COVID-19 threat appraisal and COVID-19 preventive behaviors, researchers in other countries (Barati et al., 2020; Taheri-Kharameh et al., 2020; Van Bavel et al., 2020) have indicated that not all groups in society have the same opportunity to take preventive behavioral measures such as staying home more often and avoiding meeting people beyond the same household, even when the risk assessment is high (Chen and Chen, 2020; Tanner et al., 2020). Fear of COVID-19 is positively associated with preventive behavior (H8 supported). In this case, fear of COVID-19 is assessed for both the respondent and his/her relatives. Thus, the danger of COVID-19 to certain groups of the population, and fears for the prevention of relatives can be an additional motivator for the implementation of preventive behavior (Barati et al., 2020; Parlapani et al., 2020). In addition, the results of the study show that fear of COVID-19 and engagement in COVID-19 preventive behaviors is partially mediated by COVID-19 risk appraisal (H13 supported). In the mediation model, fears of COVID-19 showed a statistically significant correlation with preventive behavior. With the addition of threat assessment as a mediator, the correlation between fears of preventive behavior became slightly weaker, but in any case remained statistically significant. The correlation between threat assessment and preventive behavior in the mediation model is also statistically significant. Thus, we can conclude that both the fear of COVID-19 and the threat assessment are important predictors of preventive behavior. Regarding socio-demographic factors, the results of the study reveal that older women, and younger people with higher education experienced a higher fear of COVID-19, while older people with higher education showed a higher risk rating (H11 partially supported), Regarding the experienced fear, the results in other studies are also ambiguous. Several studies show that younger people are inclined to experience more fear, and this fear decreases with age (Russac et al., 2007; Pasion et al., 2020). Research during the COVID-19 pandemic reveals that older people and women in particular are more afraid of COVID-19 (Adunlin et al., 2020; Hossain et al., 2020), In turn, older people rate the threat to their health higher (Shafiei and Maleksaeidi, 2020; Wu, 2020). And similar to other studies (Al-Rasheed, 2020; Banai et al., 2020; Barati et al., 2020; Khosravi, 2020), the results of our study also reveal that women and older people more frequently engage in preventive behavior (H12 supported). Even so, the specific socio-demographic variables explain a small part of the fear of COVID-19, threat appraisal and preventive behavior.

In order to test the interrelationships of TCM factors, included in the combined model, and the relationship of these factors with socio-demographic factors, hypotheses nos. 4, 9, 10, 14 were formulated within this study. The results of our study also show a correlation between trust in COVID-19 information sources and COVID-19 preventive behaviors (H9 supported). Other studies conducted during the COVID-19 pandemic also point to a positive relationship between these variables and emphasize the importance of trusting the government and other official sources of information that explain the origin of the virus, its dangers and recommendations for avoiding the disease (Al-Rasheed, 2020; Borgonovi and Pokropek, 2020). According to researchers in the TCC model, trust in the information provided by the government, the health care system and media in a crisis is fundamental. Especially in the situation where limiting the spread of the virus is the responsibility of the whole society and only through joint action is it possible to limit the further spread of the virus (Khosravi, 2020; Siegrist, 2021). It should be emphasized here that the information provided by the government during the COVID-19 pandemic and compliance with the recommended safety measures apply not only to maintaining health of the individual and avoiding the disease, but also to the health of their relatives and other members of society (Kovac et al., 2020). However, as revealed by the results of our study, this correlation is weaker than the correlation between COVID-19 threat appraisal and COVID-19 preventive behaviors. Regarding trust in COVID-19 information sources, three aspects were measured: trust in government, in news media and in the health care system. Each of these sources of information also shows a weak correlation with preventive behavior. This can be explained by the historically low level of trust of Latvian residents in public administration and news media and, according to Eurobarometer (European Union, 2019) the level of trust of the Latvian population has remained unchanged in the past year. However, there is a tendency that those members of society, who trust the information provided by the above-mentioned sources and its validity, take into account the recommendations given to limit the spread of the virus and engage in preventive behavior. The results show positive association between trust in COVID-19 information sources and threat appraisal (H4 supported). Research shows that threat assessment is closely linked to reliance on evidence-based information that clearly and accurately describes potential threats and provides recommendations for addressing them (Siegrist et al., 2003; Bamberg et al., 2020; Bäuerle et al., 2020). Information provided in a crisis is an important tool for fostering attitudes and beliefs among both individuals and society as a whole (Siegrist et al., 2003; Siegrist, 2021).

In addition, the trust in COVID-19 information sources and engagement in COVID-19 preventive behaviors are partially mediated by COVID-19 risk appraisal (H14 supported). In the mediation model, trust in COVID-19 information sources showed a statistically significant correlation with preventive behavior. If we add the threat assessment as a mediator, between trust in COVID-19 information sources and preventive behavior, the correlation becomes weaker, but remains statistically significant. At the same time, the correlation between threat assessment and preventive behavior in the mediation model is statistically significant and stronger than the correlation between trust in COVID-19 information sources and preventive behavior. Thus, we can conclude that trust in COVID-19 information sources and threat assessment are important factors predicting preventive behavior, but in terms of involvement in preventive behavior, threat assessment is more important. This means that trust in official sources of information promotes higher assessment of the virus hazards and seriousness of the situation which in turn predicts preventive behavior, as confirmed by the results of other studies (Al-Rasheed, 2020; Breakwell and Jaspal, 2020; Jørgensen et al., 2020; Khosravi, 2020; Wang et al., 2021). The results of our study also show that rural residents, higher education, and being employed indicated the highest trust in COVID-19 information sources, but in the overall model these factors explained a very small variance of trust of information sources (H10 rejected) and these results can be explained by society's overall low level of trust in the government, the health care system, and media.

The highest negative correlation appears between COVID-19 conspiracy beliefs, and trust in COVID-19 information sources (H1 supported) suggests that belief in conspiracy theories undermines trust in official sources of information and evidence-based information (Banai et al., 2020; Pummerer et al., 2021). The results also show negative correlation between COVID-19 conspiracy beliefs and COVID-19 threat appraisal (H3 supported), and as mentioned in other studies (Swami et al., 2014; Allington et al., 2020; Banai et al., 2020), our research confirms a negative correlation between COVID-19 conspiracy beliefs and COVID-19 preventive behaviors (H6 partially supported). This means that the interpretation of COVID-19 through conspiracy theories reduces the assessment of the severity of the situation and the severity of the disease, which in turn leads to the disregard of preventive behavioral measures (Kim and Kim, 2021; Pummerer et al., 2021). However, in the process of SEM (adding other independent variables in the model), this correlation was no longer significant, which reveals that explanations of COVID-19 through various conspiracies do not directly affect the individual's implementation of virus control measures.

The results of the study also reveal a positive relationship between trust in COVID-19 information sources with fear of COVID-19 (H2 supported). In the current context, where the public has no previous experience with a global pandemic of this magnitude, science-based information on the origin of the virus and its dangers, which is given to the public through the healthcare system, as well as the government and the media, can cause fear (Chang et al., 2020). Within PMT, appellate information is seen as an important stimulus for behavior modification (Brouwers and Sorrentino, 1993; Heydari et al., 2021; Kowalski and Black, 2021). In addition, in our study, fears were also assessed in relation to the health of significant others. Fears for the health of individual's family members or other close people may stimulate individuals' involvement in preventive behavior. Therefore, the information provided to the public should explain the causal links, possible risks and benefits of complying with the measures to control the virus in sufficient detail to explain the effects and risks of the virus to different groups of the public. As the results of the study revealed, fear, both directly and indirectly, through threat assessment is related to preventive behavior. Consequently, the results of other studies (Al-Rasheed, 2020; Gerritsenb, 2020; Harper et al., 2020; Mertens et al., 2020) and the results of our study also emphasize the importance of fear as a threat assessment and a contributing factor to preventive behavior.

The present survey SEM results highlight that threat appraisal (assessment of the virus hazards and seriousness of the situation) and fear are the most important factors regarding preventive behavior. The threat appraisal showed a strong relationship with preventive behavior and became a mediator between trust in COVID-19 information sources, as well as between fear of COVID-19 and preventive behavior. All variables included in the model, with the exception of belief in conspiracy theories, showed statistically significant positive correlations with preventive behavior. Belief in conspiracy theories showed a statistically significant negative correlation, but in the overall SEM model, this relationship was no longer statistically significant. These findings emphasize that public preventive messages should be very clear regarding the COVID-19 hazards. There may be a need for developing and disseminating science-based, truthful information to different groups in society, using language and an accessible approach, involving representatives from different social groups in disseminating information and communicating. It is important that the information is delivered and understood by all groups of society, thereby promoting public involvement in preventive behavior and limiting the spread of the virus.

### Ethics

The study was conducted following the principles of the World Medical Declaration of Helsinki and was approved by the Ethics Committee of Research in Riga Stradinš University (register code No. 6-1/07/4).

### Limitations

This study focuses on association of COVID-19 preventive behaviors with trust in COVID-19 information sources, COVID-19 threat appraisal, COVID-19 conspiracy beliefs and fear of COVID-19, based on a national on-line survey in Latvia. One of the potential limitations of the study is that initially potential respondents were sent invitations to participate in the study by e-mail. Therefore, it is possible that certain groups of the population were less likely to participate in the study and fill in the questionnaire than others. Another important limitation that may have influenced the results of the study is the used self-report measures. Self-report does not allow for the assessment of real behavior. Moreover, the study was cross-sectional, which does not allow for examining how (and if) the preventive behaviors of the Latvian population changed during the pandemic—nor can conclusions of causality be drawn in the examined relationships among the variables. It is also important to mention that the data were collected in July 2020, when the number of infection cases in Latvia was very small, as well as in the spring months when the prevalence of COVID-19 in other countries was very high. To test and obtain evidence for our, theoretically described and empirically tested, model it would be necessary to re-test the model with data collected over time and in countries with higher COVID-19 infection rates. Another significant limitation of the study was the fact that separate elements from TCC and PMT were used in our study to assess involvement in preventive behavior, so it was not possible to take into account factors related to preventive behavior such as effectiveness and self-efficacy. The present study also has some limitations regarding instruments. During the COVID-19 pandemic, a number of instruments have been developed, tested and widely used, potentially suitable for assessing the variables examined in this study. For example, Fear of COVID-19 Scale (Ahorsu et al., 2020; Bitan et al., 2020; Iversen et al., 2021; Magano et al., 2021). The COVID-19 Preventive Behaviors Index scale (Breakwell et al., 2021), Adolescent Conspiracy Beliefs Questionnaire (ACBQ) (Jolley et al., 2021). The Generic Conspiracist Beliefs Scale (GCBS-J) (Majima and Nakamura, 2020), Client trust in community health workers scale (CHWs) (Sripad et al., 2021). However, it should be noted here that the population of Latvia speaks Latvian or Russian and the preparatory phase of the study was limited in time, so that it was not practical to adapt and use the instruments already developed and validated in other countries. Further research is needed to verify the psychometric parameters of the reliability (e.g., test-retest reliability, important psychometric properties for instruments, which were not examined for the instruments) and validity for instruments developed in our study. The limitation is that the questionnaires were administered in two languages. It is possible that in different languages people understand some items differently, which may influence results, but in this case, we are not interested in subgroup analyses and look just for general effects, so it is not a major concern in this case. Yet another limitation is that, in SEM analysis, sum scores were used instead of latent variables. It was done because for three of five main variables, used in the model, there are only two indicators. In the future, it may be preferable, to use modified latent model with more than two indicators per variable.

## Data Availability Statement

The raw data supporting the conclusions of this article will be made available by the authors, without undue reservation.

## Ethics Statement

The studies involving human participants were reviewed and approved by Ethics Committee of Research in Riga Stradinš University (register code no. 6-1/07/4). Written informed consent for participation was not required for this study in accordance with the national legislation and the institutional requirements.

## Author Contributions

SŠ: contributed with development of the conception and design of the study; development of the questions for the questionnaire within the framework of the project “Associated factors and changes in psychological resilience, and mental preventive in the general population of Latvia during and following the COVID-19 pandemic and directions for future management and was responsible for describing the theoretical concepts, interpreting the data and preparing the manuscript. KM: contributed with development of the conception and design of the study, development of the questions for the questionnaire within the framework of the project detailed above for the first author; interpreted the data; and edited the manuscript. VP: contributed with the development of the conception and design of the study; development of the questions for the questionnaire within the framework of the project detailed above for the first author: was responsible for the translation of the developed questionnaire into Russian; was responsible for the statistical data analysis and the result part of the manuscript; interpreted the data; and edited the manuscript. JK: contributed with development of the conception and design of the study; development of the questions for the questionnaire within the framework of the project detailed above for the first author; was responsible for the translation of the developed questionnaire into Russian; and edited the manuscript. UV: contributed with data statistical analysis. AR: contributed with the development of the conception and design of the study; was responsible for the translation of the manuscript into English. JV: contributed with the development of the conception and design of the study; is national coordinator of the international study “Estimating the Effects of COVID-19 Outbreak on Mental Health.” DS was project coordinator of the international study “Estimating the Effects of COVID-19 Outbreak on Mental Health” among the general population in 42 countries. KF: principal investigator of the international study “Estimating the Effects of COVID-19 Outbreak on Mental Health” (Ahorsu et al., 2020; Latkin et al., 2021); development of the study protocol. ER: contributed with development of the conception and design of the study. All authors contributed to the manuscript and approved the submitted version.

## Conflict of Interest

The authors declare that the research was conducted in the absence of any commercial or financial relationships that could be construed as a potential conflict of interest.

## Footnotes

^1^Slimibu profilakses un kontroles centrs. Available from: https://www.spkc.gov.lv/lv/aktualitates-par-COVID-19 (Accessed April 23, 2021).

^2^Latvia: Coronavirus (COVID-19) new cases 2020 | Statista 2021 Available from: https://www.statista.com/statistics/1104735/latvia-coronavirus-COVID-19-new-cases/ (Accesed March 5, 2021).
